# In utero exposure to ultrafine particles promotes placental stress-induced programming of renin-angiotensin system-related elements in the offspring results in altered blood pressure in adult mice

**DOI:** 10.1186/s12989-019-0289-1

**Published:** 2019-01-28

**Authors:** Russell A. Morales-Rubio, Isabel Alvarado-Cruz, Natalia Manzano-León, Maria-de-los-Angeles Andrade-Oliva, Marisela Uribe-Ramirez, Betzabet Quintanilla-Vega, Álvaro Osornio-Vargas, Andrea De Vizcaya-Ruiz

**Affiliations:** 1 Departamento de Toxicología, Centro de Investigación y de Estudios Avanzados del IPN, Ciudad de México, México; 2 Departamento de Fisiología, Biofísica y Neurociencias, Centro de Investigación y de Estudios Avanzados del IPN, Ciudad de México, México; 3 Departamento de Investigación Básica, Instituto Nacional de Cancerología, Ciudad de México, México; 4 Department of Pediatrics, University of Alberta, Edmonton, Alberta Canada

**Keywords:** Ultrafine particles, Placental stress, Programming disease, Hypertension

## Abstract

**Background:**

Exposure to particulate matter (PM) is associated with an adverse intrauterine environment, which can promote adult cardiovascular disease (CVD) risk. Ultrafine particles (UFP) (small size and large surface area/mass ratio) are systemically distributed, induce inflammation and oxidative stress, and have been associated with vascular endothelial dysfunction and arterial vasoconstriction, increasing hypertension risk. Placental stress and alterations in methylation of promoter regions of renin-angiotensin system (RAS)-related elements could be involved in UFP exposure-related programming of hypertension. We investigated whether in utero UFP exposure promotes placental stress by inflammation and oxidative stress, alterations in hydroxysteroid dehydrogenase 11b-type 2 (HSD11B2) and programming of RAS-related elements, and result in altered blood pressure in adult offspring. UFP were collected from ambient air using an aerosol concentrator and physicochemically characterized. Pregnant C57BL/6J *p*^un^/*p*^un^ female mice were exposed to collected UFP (400 μg/kg accumulated dose) by intratracheal instillation and compared to control (nonexposed) and sterile H_2_O (vehicle) exposed mice. Embryo reabsorption and placental stress by measurement of the uterus, placental and fetal weights, dam serum and fetal cortisol, placental *HSD11B2* DNA methylation and protein levels, were evaluated. Polycyclic aromatic hydrocarbon (PAH) biotransformation (CYP1A1 and NQO1 (NAD(P)H dehydrogenase (quinone)1)) enzymes, inflammation and oxidative stress in placentas and fetuses were measured. Postnatal day (PND) 50 in male offspring blood pressure was measured. Methylation and protein expression of (RAS)-related elements, angiotensin II receptor type 1 (AT_1_R) and angiotensin I-converting enzyme (ACE) in fetuses and lungs of PND 50 male offspring were also assessed.

**Results:**

In utero UFP exposure induced placental stress as indicated by an increase in embryo reabsorption, decreases in the uterus, placental, and fetal weights, and *HSD11B2* hypermethylation and protein downregulation. In utero UFP exposure induced increases in the PAH-biotransforming enzymes, intrauterine oxidative damage and inflammation and stimulated programming and activation of AT_1_R and ACE, which resulted in increased blood pressure in the PND 50 male offspring.

**Conclusions:**

In utero UFP exposure promotes placental stress through inflammation and oxidative stress, and programs RAS-related elements that result in altered blood pressure in the offspring. Exposure to UFP during fetal development could influence susceptibility to CVD in adulthood.

**Electronic supplementary material:**

The online version of this article (10.1186/s12989-019-0289-1) contains supplementary material, which is available to authorized users.

## Background

Exposure to airborne particulate matter (PM_10_ and PM_2.5_) is associated with increased morbidity and mortality due to cardiovascular disease (CVD) [1, 2]. Furthermore, cardiovascular mortality from air pollution exposure could be mediated by elevated blood pressure [3]. Airborne PM includes ultrafine particles (UFP), with an aerodynamic diameter of < 0.1 μm and a high surface area to mass ratio. However, few studies have included them as a dependent factor [4] because there are no air quality regulations that address the UFP, and this class of particles is not considered to be part of the pollutants routinely monitored to assess air quality (i.e., criteria pollutants). Experimental evidence suggests that UFP may exert a higher relative toxicity than PM_10_ and PM_2.5_, because of their small size, large surface area/mass ratio, chemical composition, capacity to generate reactive oxygen species (ROS), high retention rate, penetration into deep regions of the lung, and their ability to translocate to the systemic circulation [5].

Epidemiologic and experimental studies have reported that the effects of PM exposure in adulthood may be temporary. The developmental plasticity allows the fetus to adapt to an adverse intrauterine environment, such as that induced by air pollution [6]. Several epidemiological studies have shown that exposure to PM during pregnancy is associated with detrimental developmental outcomes, such as low birth weight [7], preterm birth, and intrauterine growth restriction [8]. Interestingly, reduced birth weight has been associated with the susceptibility to hypertension [9]. Alterations in the fetal development and dysfunctional placentas have been consistently observed in experimental models of in utero exposure to PM_2.5_ [10], diesel exhaust particles (DEP) [11], or nanoparticles [12, 13] in animal models. These effects have been accompanied with intrauterine (fetus and placenta) inflammation and oxidative damage. This could be due to the direct or indirect effects of the UFP. The direct effects refer to conditions in which particles or their components, such as polycyclic aromatic hydrocarbon (PAH) and metals, traverse first the pulmonary and subsequently the placental barrier and reach the fetus, where their oxidative and inflammatory effects directly impact the fetal tissues. In contrast, the indirect effects may occur after inhaled UFP deposit in the maternal alveoli and generate ROS through their reactivity, the product of the biotransformation of PAH or any other intrinsic compounds contained in UFP, which result in oxidative stress and inflammation. The inflamed lung tissue releases inflammatory mediators (e.g., cytokines) to the bloodstream, which can reach other organs and intrauterine tissues [14]. Among the biological tools available to evaluate a transplacental exposure to pollutants is the transgenic mouse model C57BL6/6J*p*^un^/*p*^un^ strain [15], which expresses visible mutations of DNA caused by the deletion of DNA sequences in the embryo. Studies have reported a significant increase in the frequency of DNA deletions of the *p*^un^ gene in the mouse fetus, when pregnant dams were exposed to xenobiotics (DEP, TiO_2_ nanoparticles and TCDD [16–18]).

Recently, Niu et al. showed an association between the exposure to PM_2.5_ and the activation of the HPA axis [19]. The hypothalamic–pituitary–adrenal (HPA) axis regulates the production and secretion of glucocorticoids such as cortisol and corticosterone under basal and stress conditions. Excessive levels of glucocorticoids during fetal development may contribute to cardiovascular programming [20]. To protect the developing fetus from the deleterious effects of an excess of glucocorticoids, the placental enzyme HSD11B2 inactivates the glucocorticoids cortisol/corticosterone by converting them into the inactive metabolites cortisone/11b-dehydrocorticosterone [21]. Interestingly, in utero exposure to PM_10_ is associated with methylation of the promoter region of HSD11B2 [22], and furthermore, a decrease of mRNA expression and hypermethylation of the promoter region of HSD11B2 can be induced by inflammatory mediators [23] and prenatal stress [24], respectively. DNA methylation is a process that is catalyzed by the DNA methyltransferases (DNMTs), and generally, under-methylation is associated with transcription and over-expression of proteins and a hypermethylation is associated with transcriptional repression [25]. This suggests that a dysfunctional placental environment may contribute to adult cardiovascular programming, although the mechanisms of the developmental programming are not well understood.

The RAS plays a central role in the control of blood pressure and is one of the most critical steps in the pathogenesis of hypertension. The RAS components angiotensin-converting enzyme (ACE) catalyzes the conversion of the inactive decapeptide angiotensin I (Ang-I) to the active octapeptide angiotensin II (Ang-II). The action of Ang-II results from the binding to its specific receptor (AT_1_R), which mediates the vasoconstrictor response [26]. In rodents, AT_1_R exists as two distinct subtypes, AT_1_a and AT_1_b receptor (encoded by *Agtr1a* and *Agtr1b* respectively) [27]. The RAS is overexpressed during pathological cardiovascular states, such as hypertension, atherosclerosis, heart infarction, and heart failure. Moreover, in animal models, DNA hypomethylation in the promoter regions of *Agtr1b, Agtr1a* and *ACE* was also found to be correlated with hypertension [28, 29]; those studies strongly support the consequence of epigenetic modification of the hypertension programming. In addition, we have previously demonstrated that the exposure to PM (PM_2.5_ and UFP) induces the expression of RAS elements, including AT_1_R in lungs and heart [30]. Likewise, Gunnison and Chen observed a 1.5-fold increase in the differential expression of *At1r* in a lung microarray of double-knockout mice (apoE −/− and LDLr−/−) that were subchronically exposed to UFP [31].

We hypothesized that in utero exposure to UFP can promote placental stress that would result in adverse intrauterine conditions, which leads to programming hypertension from the activation of RAS-related elements. In addition, we report that in utero exposure to UFP promotes an adverse intrauterine environment that results in the susceptibility to hypertension in PND 50 male offspring.

## Methods

### Collection and physicochemical characterization of UFP

Ultrafine particles from the air of Mexico City (northern region) were collected from April to June of 2016, 5 days/week, 5 h/day (7 am - 12 pm). We used an aerosol enrichment concentrator system [32] that drew air samples that contained airborne particles through two parallel lines using > 0.25 μm cut point preimpactors to remove larger size particles. These particles are drawn through a saturation-condensation system that grows particles to 2–3 μm droplets, which are subsequently concentrated by virtual impaction. Concentrated particle suspensions were obtained by connecting the aerosol enrichment concentrator system output to a sterilized liquid impinger (BioSamplers™, SKC West, Inc., Fullerton, CA, USA) that contained ultrapure and sterile water as the collection medium. The concentration enrichment process does not alter the physical, chemical, and morphologic properties of the particles [32]. Samples from 10-weeks were pooled and stored at − 70 °C until analysis and animal exposure. We determined UFP concentration in the suspension by gravimetric analysis as previously described [33]. Briefly, after sonication of the particle suspension of the concentrated pooled sample, 50 μl aliquots were placed on sterile aluminum cuvettes to let the water evaporate during 2–4 days under constant 45% humidity and 30 °C temperature conditions. The aluminum cuvettes were weighted before and after evaporation in a microbalance to determine the mass of UFP in the suspension (used for UFP analysis and animal instillations).

We used scanning and transmission electron microscopy to assess the particle morphology and size distribution. A small drop of the particle suspension was placed on nonporous carbon tape or Lacey Formvar/carbon grid for analysis using a scanning electron microscope (SEM, Auriga 3916, Carl Zeiss) or transmission electron microscope (TEM, JEOL, JEM-ARM200F), respectively. UFP samples collected in the parallel concentrator system output using quartz were used to quantify their carbon, metal and PAH content. Elemental and organic carbon was determined by the coulombimetry method as previously described [34]. The elemental composition was analyzed using X-ray fluorescence (XRF). The X-ray detector was an Amptek (Bedford, MA, USA). The PAH species were analyzed in a gas chromatograph/mass spectrometer (6890/5973 N) (Agilent Technologies, Little Falls, CA, USA) with a quadrupole mass filter and an autosampler (model 7683). Further characterization of the UFP included hydrodynamic size (D_H_) (aggregation state of the UFP) and polydispersity index (PDI) (the broadness of the size distribution). We conducted the analysis using dynamic light scattering and Zeta potential (ζ) (dispersion stability) with laser Doppler microelectrophoresis. Both techniques were performed using a Zetasizer Nano ZS90 size analyzer (Malvern Instruments, Malvern, UK). The suspensions of the UFP concentrated pooled samples were sonicated for 5 min before use. The oxidative potential of UFP was measured on the basis of dithiothreitol (DTT) consumption as previously described in detail elsewhere [35].

### Animals and UFP in utero exposure

**Fig. 1:**
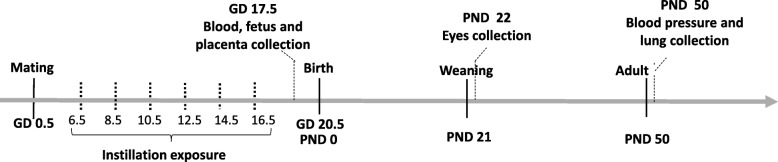
Experimental design of animal UFP exposure and sample collection. GD, Gestation day (pregnancy day); PND, post natal day (days after birth). Time mated mice were exposed by intratracheal instillation to H_2_O and UFP. The instilled dose was 12 μg/dam was distributed over six instillations on GD 6.5, 8.5,10.5, 12.5,14.5 and 16.5

Male and female C57BL/6J *p*^un^/*p*^un^ mice were kindly donated by Dr. Robert Schiestl from University of California, Los Angeles. All mice were housed in a freestanding clean room with a changing station docking port (bioBubble®, Fort Collins, Co., USA) on a 12/12 light/dark cycle in the animal facility at Cinvestav according to the institutional guidelines. All animal procedures were approved by the Internal Committee for the Use and Care of Laboratory Animals in accordance to the “Principles of Laboratory Animal Care” guidelines. Six-week-old females in estrus stage (determined by vaginal cytology as described in [36]) were mated, and the weight was monitored [37]. Once pregnancy was confirmed, each dam was housed in a separate cage until offspring birth, which remained with the dam until after weaning. The pregnant mice were exposed by intratracheal instillation as previously described in [38]. The dams were anesthetized with 4% isoflurane and instilled through the trachea with the UFP suspension (50 μl followed by 200 μl air) for the UFP exposed group (*n* = 4). For the H_2_O group, dams (*n* = 4) were instilled with sterile ultrapure water (50 μl followed by 200 μl air). A non-exposed group (no UFP, no H_2_O) was included as a control group (CTRL) (*n* = 4). The instillations were repeated six times during the fetal development after implantation on gestation day (GD) 6.5, 8.5, 10.5, 12.5, 14.5 and 16.5 day of pregnancy. The total dose per animal was 12 μg or 400 μg/kg (Fig. 1).

### Tissue collection

On GD 17.5, after the last UFP instillation, the dams were anesthetized with 4% isoflurane and euthanized by cervical dislocation. For the prenatal study, the uterus (*n* = 4 per group) of each dam was collected, and visual evaluation was carried out to detect and quantify embryo reabsorption and to evaluate the fetal and placental effects of the in utero exposure to UFP. After removal of the uterus (that included placenta, amnion, fetus, and membranes), placentas and fetuses were weighed, analyzed for weight difference and averaged per litter. The fetuses and placentas were quick-frozen in liquid nitrogen and stored at − 70 °C until analysis. To perform the placenta and fetus analyses of biotransformation enzymes, oxidative damage, and HSD11B2 methylation and protein levels in (*n* = 6 per group), both tissues were mixed and randomly selected (see the sex ratio Additional file 1: Table S2). Also, the lung and total blood of dams (obtained by cardiac puncture) were obtained to evaluate biotransformation enzyme, serum cortisol and systemic inflammation. For postnatal analysis (*n* = 6 per group), dams were exposed as described previously, and they were allowed to deliver spontaneously. The total litter size and the number of live pups were recorded on postnatal day 0 (PND 0). On PND 21 or after weaning, the offspring were euthanized. Their eyes were extracted for retinal pigment epithelium (RPE) test; we analyzed 15 pairs of eyes of 15 (30 eyes per group), regardless of offspring sex. Only male offspring were allowed to grow into 50 PND to measure the blood pressure, collect the lungs for methylation analysis and protein levels of RAS-related elements (*n* = 4 per group). Our study focused on male offspring to control for cyclic hormonal variation. The placentas and fetuses were mixed and randomly selected for analysis.

### Deletion/eyespot assay in *p*^un^ mice

To evaluate the transplacental in vivo exposure we used a homozygous mouse model (C57BL/6J *p*^un^/*p*^un^), taking advantage of the *p* (un) allele (*pink-eyed unstable*), which contains a tandem duplication of a 70-kb fragment spanning exons 6 to 18 of the p gene in mice. Mice with the *p* (un) allele have a transparent RPE. A deletion of these duplications induced by the exposure to xenobiotics restores the p gene, which encodes a protein responsible for the assembly of a black-colored melanin complex that will produce black pigmented cells (eye-spots) in the offspring. The eyes extracted from the weaned offspring were dissected to display the RPE for the deletion/eyespot assay as described previously in [39].

### Serum and fetus cortisol determination

Mice and rats, which are nocturnal, are commonly used in glucocorticoid research, and exhibit an inverse tempo in glucocorticoid profile, with peak plasma levels of corticosterone and cortisol at night and decreased concentrations during the day [40]. Also, although corticosterone is the main glucocorticoid involved in the regulation of stress responses in rodents, several studies have shown increased cortisol levels in plasma and adrenal glands of mice following environmental stress [41, 42]. For these reasons and to minimize serum cortisol variations, dams were always euthanized during the day (between 11:00 am and 2:00 pm), and cortisol was chosen over corticosterone to determine glucocorticoid levels. To evaluate the placental dysfunction and activation of the hypothalamic pituitary adrenal axis (HPA) induced by the in utero exposure to UFP, the cortisol levels of the homogenized fetuses and dam sera were measured using a Mouse/Rat cortisol ELISA kit (Sigma-Aldrich, Poole, Dorset UK) following the manufacturer’s instructions. To obtain the total protein, the whole fetuses were homogenized with Nonidet-P40 buffer (150 mM NaCl, 1% NP40, 50 mM Tris–HCl, pH 8.0, and protease inhibitors) and centrifuged at 10,000 rpm/4 °C, and the supernatant was collected.

### Detection of placental and fetus 8-OH-dG

The levels of 8-hydroxydeoxyguanosine (8-OHdG) were assessed in the placental and fetal DNA to evaluate the oxidative damage produced by the UFP. The DNAzol reagent (Molecular Research Center, Cincinnati, OH, USA) was used to isolate genomic DNA of whole placentas and fetuses (as previously described). An 8-OH-dG ELISA kit (Cayman Chemical, Ann Arbor, MI, USA) was used, and all procedures were conducted according to the manufacturer’s instructions.

### Analysis of cytokines and chemokines

The pro-inflammatory cytokines interleukin-β (IL-β), IL-6 and tumor necrosis factor-α (TNF-α), the anti-inflammatory cytokines IL-10 and IL-4, chemokines including neutrophil chemoattractant (KC), macrophage inflammatory protein 2 (MIP-2) and monocyte chemoattractant protein 1 (MCP-1), and vascular endothelial growth factor (VEGF) were measured in the dam sera and the total protein of the dam lungs, placentas and fetuses using an MCYTOMAG-70 K Milliplex MAP Mouse Cytokine/Chemokine magnetic bead panel (EMD Millipore, Billerica, MA, USA) following the manufacturer’s instructions.

### Western blot

Frozen tissues were homogenized in Nonidet-P40 buffer as described above to extract the total protein. The protein was quantified using the Bradford protein assay, then 30 μg of the protein was electrophoresed (SDS-PAGE), and then transferred to nitrocellulose membranes. The membranes were blocked for 1 h with 5% of not-fat milk in PBS. The membranes were then incubated overnight with the primary antibodies to HSD11B2 (1:300; rabbit polyclonal, ab115696, Abcam, Cambridge, MA, USA), NQAO1 (1:500; rabbit polyclonal, ab34173, Abcam), AT_1_R (1:500; rabbit polyclonal, Sc-579, Santa Cruz Biotechnology, Santa Cruz, CA, USA), ACE (1:1000; goat polyclonal, Sc-12,184, Santa Cruz Biotechnology) or CYP1A1 (1:500; mouse polyclonal, Sc-253,041, Santa Cruz Biotechnology), and GAPDH (1:1000, mouse monoclonal, Sc-32,233, Santa Cruz Biotechnology) was used as a loading control. Horseradish peroxidase (HRP)-conjugated secondary antibody (1:10000; Bio-Rad Laboratories, Hercules, CA, USA) was incubated with the membranes at room temperature for 1 h, and the HRP was subsequently detected using the Luminata Forte Western HRP substrate reagent (Millipore, Burlington, MA, USA). The expression levels were visualized by exposure to X-ray film and quantified by optical densitometry, using the ImageJ software (free from the National Institutes of Health).

### DNA methylation analysis

Placentas, fetuses, and lung samples of the male offspring at PND 50 were obtained, and the DNA was extracted and purified using the Genomic DNA Purification Kit (DNeasy blood and tissue, Qiagen, Hilden, Germany). Bisulfite conversion was performed on 1 μg of genomic DNA using the EZ-96 DNA methylation kit (Zymo Research, Orange, CA, USA) according to the manufacturer’s instructions. Tissue-gene specific methylation in the promoter region was assessed for *HSD11B* (9 CpG sites), *Ace* (7 CpG sites)*, Agtr1a* (4 CpG sites), and *Agtr1b* (5 CpG sites) genes. The primer design was focused on CpG dinucleotides located in the CpG island closest to the transcription start site (TSS), on the basis that H3K27Ac indicates as high a probability of transcription factor binding as CTCF. Polymorphisms and repetitive elements were avoided, and the UCSC database and MethPrimer tool were used [43, 44]. DNA methylation of *Ace, Agtr1a* and *Agtr1b* was analyzed in fetal samples and those from the lungs of the male offspring at PND 50, and methylation of *HSD11B* gene was evaluated in the placenta*.* After bisulfite treatment, the samples were amplified by PCR. A 30-μl PCR was carried out using 15 μl of GoTaq Hot Start Green Master Mix (Promega, Madison, WI, USA) with 10 pmol of each primer (Sigma Aldrich) and 1 μl of the bisulfite-treated DNA. The PCR and sequencing primers are shown in Additional file 2: Table S1. Two sequencing primers were used to analyze the total CpG sites for *HSD11B* and *ACE*. Pyrosequencing analysis was done using the PyroMark® Q24 Pyrosequencing System with PyroMark Gold reagents (Qiagen, Hilde, Germany) according to the manufacturer’s instructions. PyroMark® Control Oligo, which contains 50% 5-mC and 50% cytosine, was used. In addition, several thymine residues were intentionally added as controls to verify the bisulfite conversion of each run. The samples were run in duplicate. The methylation values were quantified using the PyroMark® Analysis software version 2.0.7 (Qiagen, Hilde, Germany). The output from pyrosequencing analysis is reported as a percent of 5-methylcytosine (% 5-mC) at each CpG site.

### Blood pressure

Blood pressure was measured in the male offspring at PND 50 using the tail-cuff pressure system (Digital pressure meter LE 5002 LETICA, Panlab, S.L. Barcelona, Spain). The mice were acclimated to the restraint and warm-up procedures during the week before the blood pressure measurements. During the acclimation days, the male mice were placed into a restraining tube used for the tail-cuff and warmed-up in a hot chamber at 35 °C for 10 min to vasodilate the blood vessels. After the acclimatization period, the mice were restrained and preheated for 10 min, after which the blood pressure was measured using a fitted cuff with a pneumatic pulse sensor. At least ten consecutive blood pressure values were recorded per mouse and averaged.

### Statistical analysis

All data are expressed as the means ± SEM or SD. One-way or two-way ANOVA with Tukey’s *posthoc* test was used to perform the statistical tests. Only for the analysis of the number of dams with resorbed embryo and number of resorbed embryos the Student’s t-test was performed. The results were considered statistically significant at *p* < 0.05. GraphPad Prism (GraphPad Software, San Diego, CA, USA) was used for the statistical analysis.

## Results

### Physicochemical characterization of the collected UFP

**Fig. 2:**
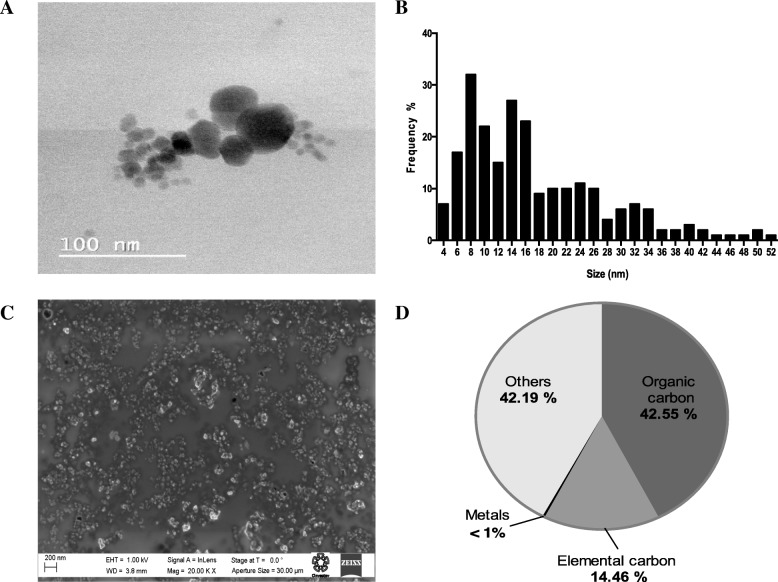
Physicochemical characterization of UFP. The geometric size was determined by **a**) TEM imaging, **b**) the frequency of the size distribution of UFP, **c**) SEM imaging, and **d**) fractional composition of the UFP collected

Ultrafine particles collected from the air pollution were characterized dry and in suspension (distilled, sterilized water) (Fig. 2a-c and Table 1). Observations by transmission electron microscopy and scanning electron microscopy showed that the UFP possessed diameters smaller than 60 nm and had a rounded shape. When suspended in H_2_O, the particles demonstrated a hydrodynamic diameter of 94 nm, a polydispersity index of 0.632 and a Zeta potential of 28 mV, which indicated a stable dispersion in H_2_O. The UFP suspension showed a high redox potential (0.174 nmol DTT/min*μg). Measurements of the UFP suspension mass by gravimetrical analysis showed a concentration of 2 μg of particles in 50 μl of suspension. The UFP chemical composition was 42.55% organic carbon (OC), 14.46% elemental carbon (EC), < 1% metals, and 42.19% other compounds (Fig. 2 d). The most abundant metals were Fe, K, Ca, Cr, Ni, Zn, Cu and Ti, and the most abundant PAH species were naphthalene, phenanthrene and benzo[a]pyrene (Table 2).

**Table 1:** Characterization of UFP dispersion in H_2_O

D_H_ (nm)	PDI	ζ (mV)	Redox potential (nmolDTT/min*μg)
94 ± 8.65	0.632 ± 0.17	28 ± 0.03	0.174 ± 0.002

D_H_: Hydrodynamic diameter. PDI: Polydispersion index. ζ: zeta potential. DTT: Dithiothreitol

The UFP pool in H_2_O at 40 μg/ml, were analyzed in triplicate. Values represent mean ± SEM

**Table 2:** Chemical composition of UFP. Values are representative of *n* = 6 filters and are given as ng/μg of UFP

Metals	mean	SD
K	67.109	± 10.383
Ca	56.110	± 8.703
Ti	12.760	± 9.086
Cr	50.667	± 1.589
Fe	88.053	± 10.242
Ni	50.881	± 29.497
Cu	23.717	± 13.757
Zn	40.786	± 20.957
PAH species	mean	SD
Naphtalene	4.757	± 2.703
Acenaphthylene	1.077	± 2.206
Fluorene	1.597	± 2.465
Phenanthrene	3.090	± 3.600
Anthracene	1.585	± 1.532
Fluoranthene	0.899	± 1.284
Pyrene	0.934	± 0.630
Retene	0.938	± 0.887
Benzo [a] anthracene	0.611	± 0.511
Crisene	0.994	± 0.933
Cyclopenta [c, d] pyrene	0.628	± 0.703
5-Methylcrisene	0.243	± 0.170
Benzo [b] fluoranthene	0.785	± 0.875
Benzo [k] fluoranthene	0.519	± 0.581
Benzo [j] fluoranthene	0.459	± 0.492
Dibenzo [a, e] pyrene	0.031	± 0.071
Benzo [e] pyrene	0.893	± 1.002
Benzo [a] pyrene	3.160	± 1.497
Indeno[1,2,3-cd] pyrene	1.192	± 0.606
Benzo [ghi] perylene	1.727	± 1.022

### Transplacental in vivo UFP exposure does not induce DNA deletion

The deletion/eyespot assay in C57BL/6J *p*^un^/*p*^un^ mice was used to evaluate deletion events as a result of the transplacental in vivo UFP exposure. The numbers of eyespots or DNA deletions per RPE of weaning offspring were quantified (Table 3). The UFP-treated mice showed 1.9 ± 0.16 eyespots per pup, and there were 1.7 ± 0.17 in the H_2_O-exposed mice and 1.4 ± 0.13 in the CTRL mice. There were no significant differences among the groups. Therefore, in utero UFP exposure did not induce an increase in DNA deletions in RPE in the *p*^un^ model.

**Table 3:** Frequency of DNA deletions as an evaluation of transplacental in vivo UFP exposure

	No. RPE^a^	Average number of eye-spots per RPE ± SEM	Average number of cells per eye-spot ± SEM
CTRL	30	4.9 ± 0.17	2.3 ± 0.14
H_2_O	30	4.6 ± 0.13	1.9 ± 0.40
UFP	30	5.1 ± 0.16	2.1 ± 0.09

^a^One RPE corresponds to one eye

### In utero UFP exposure induces embryonic reabsorptions and decreases fetal and extraembryonic tissue weight

Table 4 shows the dam weights measured at GD 17.5; a significant decrease in the body weight gains of the dams that were exposed to UFP was observed compared to the H_2_O-exposed and CTRL groups. Each uterus was extracted and examined for embryo reabsorptions, and we identified a significant increase in the number of dams with resorbed embryos as well as in the number of resorbed embryos per mouse after the exposure to UFP compared to H_2_O-exposed and CTRL groups. The individual placentas and fetuses were weighed, and their weights were significantly lower in mice that were exposed to UFP compared to the H_2_O-exposed and CTRL groups. Another group of dams delivered spontaneously (PND 0) and the total litter size was significantly decreased after the UFP exposure versus the H_2_O-exposed and CTRL groups.

**Table 4:** Embryonic resorptions, fetal and extra-embryonic tissues weight

	CTRL (*n* = 4)	H_2_O (n = 4)	UFP (n = 4)
Litter size (mean ± SEM)	7.25 ± 0.47	8.50 ± 0.64	5.50 ± 0.69*
No. dams with resorbed embryo	1	1	3^+^
No. resorbed embryo	1	2	7^++^
Dam weight on GD 17.5 (g) (mean ± SEM)	34.3 ± 0.90	35.73 ± 1.63	32.7 ± 0.76*
Uterus weight on GD 17.5 (g) (mean ± SEM)	9.59 ± 0.48	10.33 ± 0.80	7.98 ± 0.81
Fetus weight on GD 17.5 (g) (mean ± SEM)	0.90 ± 0.02	0.94 ± 0.05	0.79 ± 0.04*
Placenta weight on GD 17.5 (g) (mean ± SEM)	0.14 ± 0.003	0.14 ± 0.001	0.12 ± 0.001*

* Statistically significant difference compared to the control and H_2_O group * *p* < 0.05. One-way ANOVA

+ Statistically significant difference compared to the control and H_2_O group + *p* < 0.05 and ++ *p* < 0.05. T-Student

**Table 5:** Blood pressure in male 50 PND offspring

	CTRL (*n* = 6)	H_2_O (n = 6)	UFP (n = 6)
SAP (mm Hg)	136.6 ± 3.12	141.9 ± 5.36	156.4 ± 3.90 *
DAP (mm Hg)	109.1 ± 3.33	106.7 ± 7.54	124.2 ± 2.80*
MAP (mm Hg)	116.0 ± 3.35	110.1 ± 6.98	133.9 ± 4.77*
HR (bts/min)	614.4 ± 18.24	608.4 ± 30.88	614.8 ± 21.10

Mean ± SEM of values of systolic arterial pressure (SAP), diastolic arterial pressure (DAP), mean arterial pressure (MAP) and heart rate.

* Statistically significant difference compared to the control and H_2_O group * *p* < 0.05. One-way ANOVA

### In utero exposure to UFP induces placental dysfunction modulation and promotes activation of hypothalamic-pituitary-adrenal axis (HPA)

**Fig. 3:**
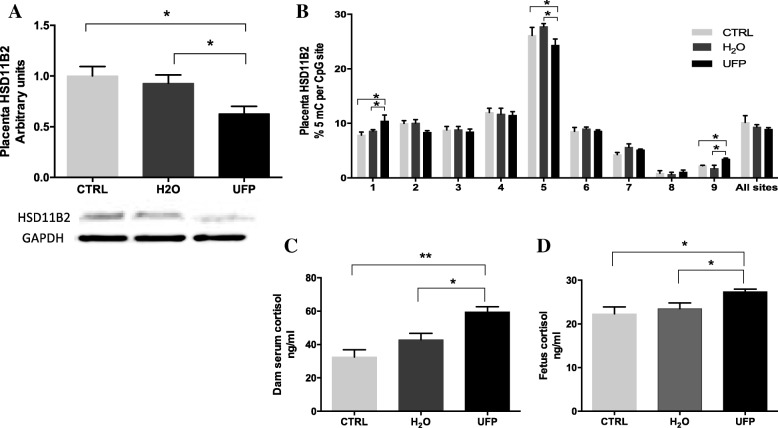
In utero exposure to UFP induces placental dysfunction and promotes activation of hypothalamic pituitary adrenal axis (HPA). **a** Protein levels and representative image of Western blot of placenta HSD11B2. n = 6/group; * *p* < 0.05. One-way ANOVA. **b** DNA methylation of the HSD11B2 promoter in placenta determined by pyrosequencing. *n* = 4/group; * p < 0.05. Two-way ANOVA. **c** Dam serum cortisol and **d** fetus cortisol were determined by ELISA. n = 4/group. Data are expressed as mean ± SEM. *p < 0.05 and ** *p* < 0.01. One-way ANOVA

To evaluate the effect on placental stress that was caused by in utero exposure to UFP, we measured the protein levels and methylation of the promoter region of HSD11B2 (Fig. 3a and b). The protein level of HSD11B2 was statistically lower in the placentas from dams that were exposed to UFP compared to the H_2_O-exposed and CTRL groups. Likewise, the methylation of 9 CpG sites of its promoter region was evaluated by pyrosequencing. A significant increase in the %5-mC in the first and ninth CpG sites was observed in the placentas of the dams that were exposed to UFP compared to the H_2_O-exposed and CTRL groups. In contrast, in the fifth CpG site, a significant decrease in the %5-mC was observed in the UFP-exposed group compared to both control groups. In addition, the mean of the %5-mC in all CpG sites was not different among groups.

On the other hand, several studies have suggested that the HPA may play a critical role in developmental programming [20]. To evaluate whether HPA activation in pregnant dams that were exposed to UFP promoted a deficit in placental HSD11B2, we measured the levels of cortisol in the dam and fetal serum on GD 17.5. Figure 3 c and d show that in utero exposure to UFP significantly increased the serum cortisol levels in the dams and fetuses compared to the respective control groups.

### In utero exposure to UFP induces oxidant damage

**Fig. 4:**
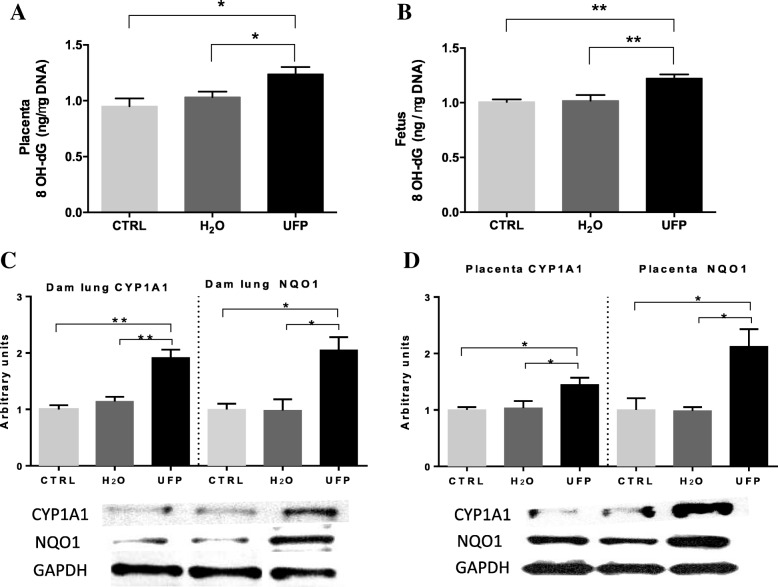
In utero exposure to UFP induces oxidant damage by metabolism of PAHs. Levels of 8-hydroxydeoxyguanosine (8-OHdG) were measured by ELISA in **a**) placenta and **b**) fetus. *n *= 4/group; * *p* < 0.05 and ** *p* < 0.01. One-way ANOVA. **c** Protein levels and representative image of Western blot of dam lung CYP1A1 and NQO1, and **d**) placental CYP1A1 and NQO1. *n* = 4/group. Data are expressed as mean ± SEM. * *p* < 0.05 and ** *p* < 0.01. One-way ANOVA

The ultrafine particles have higher redox activity than larger particles due in part to their high content of organic compounds such as PAH. These compounds induce oxidative stress through their biotransformation by the cytochrome P450 CYP1A1 isoform and NQO1. This process generates redox-active quinones, which can lead to the formation of 8-hydroxydeoxyguanosine (8-OHdG). Therefore, we investigated whether in utero UFP exposure promoted oxidative damage to the placentas and fetuses through the PAH metabolism. ELISAs were used to measure the levels of 8-OHdG in the placentas and fetuses (Fig. 4a and b) as a marker of oxidative damage. The in utero UFP exposure promoted oxidative damage as indicated by a significant increase in 8-OHdG in both in placentas and fetuses. The CYP1A1 and NQO1 protein levels significantly increased in both the dam lungs and placentas in the UFP-exposed group compared with the control groups (Fig. 4c and d).

### In utero exposure to UFP induces systemic and intrauterine inflammation

**Fig. 5:**
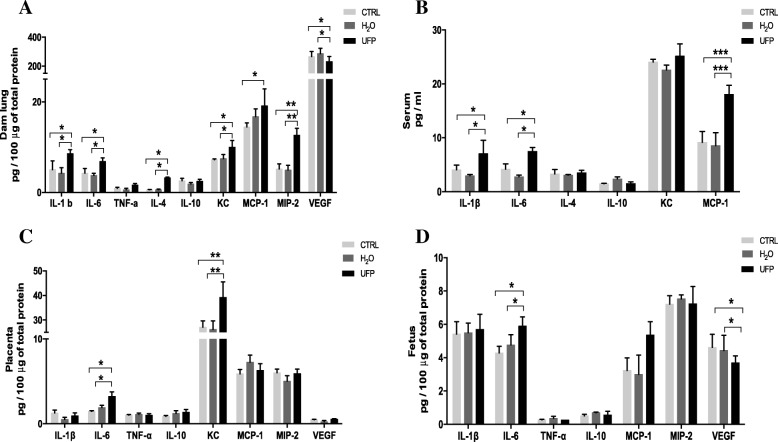
In utero exposure to UFP induces systemic and intrauterine inflammation. Concentrations of pro-inflammatory cytokine: interleukin-β (IL-β), IL-6 and tumor necrosis factor-α (TNF-α). The anti-inflammatory cytokines IL-10 and IL-4. Chemokines: neutrophil chemoattractant (KC), macrophage inflammatory protein 2 (MIP-2) and monocyte chemoattractant protein 1 (MCP-1). Vascular endothelial growth factor (VEGF) ware measured with multiplex magnetic beads in **a**) dam serum collected, **b**) dam lung, **c**) placenta and **d**) fetus total protein. *n* = 4/group. Data are expressed as mean ± SEM. *p < 0.05, ** *p* < 0.01 and *** *p* < 0.001. One-way ANOVA

To determine whether in utero UFP exposure induced systemic and intrauterine inflammation, pro-inflammatory and anti-inflammatory cytokines and chemokines were measured in dam serum, dam lung, placenta and fetus total protein. All studied cytokines and chemokines were detected in dam lungs (Fig. 5a), and elevated concentrations of IL-β, IL-6, IL-4 KC, MCP-1 and MIP-2 were observed in the UFP-exposed group. Furthermore, in the dam sera (Fig. 5b) only IL-1β, IL-6 and MCP-1 were found to be increased in the UFP-exposed group (Fig. 5b). On the other hand, increased levels of IL-6 and KC were consistently induced by in utero UFP exposure in the placentas and fetuses (Fig. 5c and d). Conversely, fetal VEFG showed a significant decrease.

### In utero exposure to UFP induces cardiovascular programming by DNA hypomethylation of RAS-related elements AT_1_a, AT_1_b receptor and ACE promoters in the fetuses and lungs of the male offspring AT PND 50

**Fig. 6:**
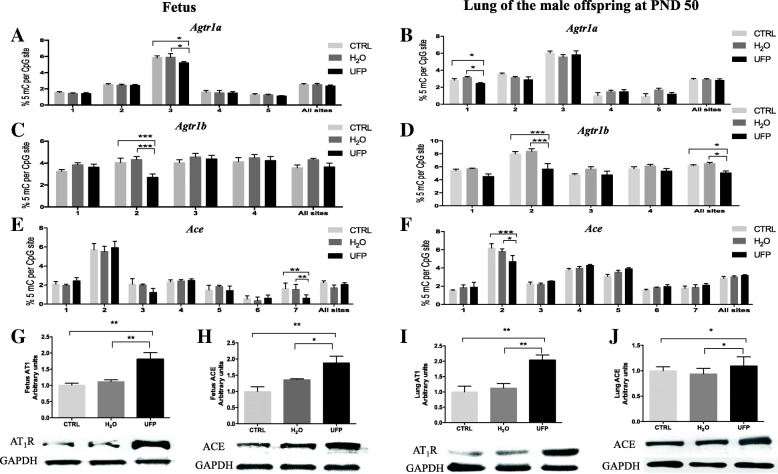
In utero exposure to UFP induces cardiovascular programming by DNA hypomethylation of the *Agtr1*a, *Agtr1b* and *ACE* promoter in fetus and adult lung. Percentage of methylated 5 CpG sites in *Agtr1a*, *Agtr1b* and *ACE* promoter in the **a**, **c** and **e**) fetus and **b**, **d** and **f**) lung. *n* = 4/group; *p < 0.05, **p < 0.01 and *** *p* < 0.001. Two-way ANOVA. **c** Protein levels and representative image of Western blot of G) fetus AT_1_R and H) ACE, and I) lung AT_1_ and ACE. *n* = 4/group. Data are expressed as mean ± SEM. * *p* < 0.05 and ** *p* < 0.01. One-way ANOVA

Fetal programming of hypertension could be mediated by an epigenetic mechanism, through DNA methylation of the promoter region. RAS has been implicated in the pathogenesis of hypertension. Furthermore, components of RAS, similar to the angiotensin receptors *Agtr1a* and *Agtr1a,* and *Ace* are subjected to methylation [28, 29]. Therefore, to evaluate whether in utero UFP exposure induced changes in the methylation pattern in the promoter regions of the RAS genes, we assessed the percent of 5-mC at each CpG site in the promoter regions of *Agtr1a, Agtr1b* and *Ace* genes of the fetuses and lungs of the male offspring at 50 PND. Five CpG sites were evaluated for the *Agtr1a* promoter region, and a significant difference in the third CpG site was observed in the UFP-exposed fetuses (Fig. 6a) whereas in the lungs of the male offspring at PND 50 (Fig. 6b), a significant change was observed in the first CpG. The percent of 5-mC in the second CpG site of the *Agtr1b* promoter region was significantly lower in the fetuses (Fig. 6c) as well as in the lungs of the male offspring at PND 50 (Fig. 6d) in the UFP-exposed mice compared with the H_2_O-exposed and CRL groups. Additionally, Fig. 6d exhibited that in utero UFP exposure significantly decreased the average methylation of all sites in the lungs of the male offspring at PND 50. Of the 7 CpG sites analyzed in the *Ace* promotor region, only the seventh and second CpG sites were significantly lower in the fetuses and lungs of the male offspring at PND 50 (Fig. 6e and f), respectively, in UFP-treated animals compared with both control groups.

Hypomethylation is associated with transcriptional activation and the overexpression of proteins. To confirm these changes, we assessed the protein levels of AT_1_R and ACE in the fetuses and lungs of the male offspring at PND 50 by western blotting. We used an antibody that recognizes both AT_1_a and AT_1_b receptors, which are highly homologous (95% identical in their amino acid sequences) [45]. In the fetuses, significant increases in AT_1_R and ACE were induced by the in utero UFP exposure (Fig. 6g and h). Additionally, significant differences were observed in the AT_1_R and ACE protein levels in the lungs of the male offspring at PND 50 in the in utero UFP exposed groups compared with the H_2_O-exposed and CTRL groups.

### In utero exposure to UFP promotes altered blood pressure in the male offspring at PND 50

We investigated whether in utero UFP exposure induced long-term effects on blood pressure in offspring at PND 50 (Table 5). Systolic and mean arterial pressure were statistically increased in the offspring male exposed in utero to UFP compared to both control groups whereas the diastolic pressure was significantly higher only compared with the CTRL group. No significant differences were observed in the heart rate.

## Discussion

There is substantial evidence that intrauterine stress induced by environmental stressors may program the later development of diseases. Of special interest for this study was the association of cardiovascular disease-hypertension with exposure to ambient PM. Experimental evidence suggests that UFP could pose a greater risk to human health than PM_10_ and PM_2.5_, given their smaller size, large surface area/mass ratio, and increased reactivity. We, therefore, developed a stable dispersion of the UFP to expose pregnant mice through intratracheal instillation. Our results indicate that the exposure to UFP during fetal development (12 μg/animal or 400 μg /kg) promoted placental stress and gene programming associated with toxicological factors of mouse hypertension. In this study, we exposed to 12 μg of UFP per dam, distributed every 2 days 6 times during the fetal development stage. The exposure of 2 μg/m^3^ of UFP in the mice model is equivalent to 7–86 μg/m^3^ in PM_2.5_ in mice and 29–86 μg/m^3^ in humans, which is a dose representative to a human exposure in a highly polluted city according to the World Health Organization -*Exposure to ambient air pollution from particulate matter report* [46] and based on the study of Gavett et al. [47].

The reduction of the dams’ weight at GD17.5 in the UFP exposed group could be due to a lesser physiological weight gain during pregnancy and the lower litter size (Additional file 1: Table S2). Also, promoted placental stress as manifested by decreased weight in the fetus at GD 17.5, an increased number of resorbed embryos, intrauterine inflammation and oxidative damage. The exposure also induced the HPA axis as indicated by increases in the cortisol levels in the dam and fetal sera, decreased dam weights and decreased protein levels and hypermethylation in two CpG sites of the promoter region of the *HSD11B2*. PND 50 male offspring demonstrated altered blood pressure, possibly triggered by RAS-related elements; we observed increased protein and hypomethylation levels in the promoter region of ACE and AT_1_R. These findings are consistent with the findings of Barker et al. [6], who described the link between intrauterine adversity by environmental stressors with an increased risk of CVD in adulthood.

In this study, we focused on the in utero exposure to UFP and observed a significant decrease in placenta and fetal weights at GD 17.5: low birth weight is one of the main effects observed from in utero PM exposure [48, 49] and has been used as a marker of an adverse intrauterine environment. Also, we observed that placental weight was also decreased as a result of the exposure to UFP. Although there are few experimental findings after PM exposure on the placenta and fetal weight, our findings are consistent with other published results. Exposure to diesel exhaust particles (DEP), resulted in a decreased placental weight in pregnant female C57Bl/6J mice exposed to 300 μg/m^3^ DEP [11], and in a reduction in the offspring body weight of C57BL/6BomTac after exposure of the pregnant mice to 19 mg/m^3^ DEP [50]. Low birth weight is also associated with an increased risk of subsequent adult diseases, including hypertension, and other cardiovascular diseases [51, 52]. Furthermore, we observed that placental weight was decreased by the in utero UFP exposure. Epidemiological evidence has shown that the weight and specific features of placental development are related to hypertension, the risk of cardiac death, and the estimated risk of death from coronary artery disease in the offspring in later life [53, 54]. The placenta is critical for normal fetal growth and development. However, a dysfunction in its homeostasis is responsible for triggering mechanisms related to CVD at adulthood [55, 56]. Here, we observed placental dysfunction due to the in utero exposure to UFP and possibly related to high cortisol levels which may have resulted from a decrease in the protein expression of HSD11B2. The placental enzyme HSD11B2 inactivates glucocorticoids and thus protects the developing fetus from the deleterious effects of excess glucocorticoids [21]. Due to the increase of HPA axis activity, which had the consequence of an increase in the dam’s serum cortisol, and a reduction of placental HSD11B2 protein expression after in utero UFP exposure, the fetal level of cortisol was also increased. Epidemiological studies have shown similar effects on the serum levels of cortisol due to the activation of the HPA axis after the exposure to PM_2.5_ [22]. Although corticosterone is considered to be the main glucocorticoid involved in the regulation of stress responses in rodents, several studies have shown increased cortisol levels in plasma and adrenal glands of mice following environmental stress [41, 42]. Moreover, animal studies have shown that prenatal glucocorticoid excess that resulted from maternal stress or due to exogenous administration to the mother or the fetus was associated with gene programming effects of the cardiovascular system [57, 58]. The mechanisms by which glucocorticoids mediate these effects are unclear but may include epigenetic modifications. Interestingly, our data indicated that the in utero exposure to UFP significantly increased the *HSD11B2* promoter methylation, which resulted in the decrease of HSD11B2 protein levels and high cortisol levels in fetuses. The increased methylation of *HSD11B2* promoter is consistent with a previous study in a human population that showed that exposure to PM_10_ during early pregnancy was associated with *HSD11B2* placental DNA methylation [23] and other genes [59].

We collected UFP of air from Mexico City, and according to the physicochemical characteristics such as rounded shape, the proportion of the components (OC>EC>others> metals), and high potential redox activity, we propose that the UFP came from anthropogenic sources of fuel burning, in accordance with the predominant sources in the area (heavy traffic and industry). Moreover, oxidative stress and inflammation have been well confirmed to induce stress and may potentially be involved in the perturbation of pregnancy and developmental toxicity. In the lungs of dams that were exposed to UFP, a significant increase in the 8-OHdG levels was observed, as well as an increase in the protein levels of CYP1A1 and NQO1: these enzymes induce ROS through the biotransformation of PAH (which are present in the UFP). On the other hand, we demonstrated pulmonary inflammation by the increase of pro-inflammatory and anti-inflammatory cytokines and chemokines. The increase in ROS generation, which was probably the result of PAH biotransformation, could increase pro-inflammatory cytokines, potentially through the activation of nuclear factor kappa B (NF-kΒ). Consistently, animal studies have shown increased pulmonary inflammation after intratracheal instillation of ultrafine DEP [60, 61]. In addition, we observed systemic inflammation and a release of cytokines from the lung into the systemic circulation as demonstrated by increases in IL-1β and IL-6 in the dam sera. Inflammatory mediators derived from systemic circulation may be transported to the intrauterine environment, the dam’s essential organs and to fetal tissues, thus inducing placental stress. Several constituents of the particles, such as PAH and metals, when present in the bloodstream can be factors that cause the inflammatory process. Additionally, the observed HPA axis activation could be stimulated by pro-inflammatory cytokines, such as IL-1β, TNFα and IL-6, as reported by John & Buckingham [62]. The biotransformation of PAH in the placenta and fetus could affect the expression of immunoregulatory genes, which suggests that the chronic activation of the AhR would lead to elevated IL-6, as indicated by various studies that have shown cross-talk between AhR and NF-kB [63]. We confirmed an increase in oxidative damage and IL-6 production in the exposed fetuses from this study. Surprisingly, the levels of vascular endothelial growth factor (VEGF) in the fetuses were decreased, an event that has been associated with feto-placental vascular dysfunction [64]. VEGF is an endothelial cell-specific mitogen that promotes placental vascularization, studies have reported a decrease in VEGF during preeclampsia [65].

According to our findings, in utero UFP exposure induced oxidative damage and inflammation in the placentas and fetuses. However, contrary to our expectations, the *p*^un^ model mice transplacentally exposed to UFP showed a no significant increment in the levels of DNA deletions in RPE. It is possible that the UFP dose (70 μg/kg/day) used in this study was not enough to induce detectable DNA deletions. Previous studies reporting the deletion of the *p*^un^ gene after exposure to DEP and TiO_2_ nanoparticles during gestation used much higher doses (500 mg/kg/day) [16, 17].

Finally, we suggest that the suboptimal environment generated by the UFP exposure can affect the programming of CVD. Developmental programming, or developmental origins of health and disease, refers to the changes in fetal structure and physiology that result from adverse early life exposures that lead to increased disease risk in later life [66]. Programmed characteristics are believed to be largely stable after the postnatal period and have been associated with increased risk of coronary heart disease, type 2 diabetes, stroke and hypertension [6]. Stressors that lead to fetal programming have been identified and include nutritional factors such as over- and undernutrition [67], high glucocorticoid exposure and environmental pollutants [68]. However, results regarding the developmental origins of health and diseases (DOHaD) have been associated, later in life, with an increased risk of cardiovascular pathologies, such as hypertension [5]. In this regard, the RAS is strongly involved in blood pressure regulation and cardiovascular disease programming; hence, the overexpression of ACE and AT_1_R in the lung may induce hypertension. Our findings showed that in utero UFP exposure significantly decreased the %5-mC in the *Ace*, *Agtr1a,* and *Agtr1a* promoter regions in the fetuses and lungs of the male offspring at PND 50. The expression of the ACE and AT_1_R proteins in the fetuses and lungs of the male offspring correlated well with the DNA methylation levels. Methylation alterations in RAS-related genes, such as *Ace*, *Agtr1a*, and *Agtr1b,* have been shown to play a role in the fetal programming of hypertension [69–71]. Hence, the increased blood pressure in the offspring at PND 50 was consistent with the over-expression of components of the RAS in the lung. Although AT_1_a is expressed in all tissues, AT_1_b is expressed only in the adrenal glands. A previous study by Goodson et al. [72] adds support to the finding that in utero exposure to PM promotes susceptibility to cardiovascular disease, inasmuch as they observed differential methylation of a gene identified as likely playing an important role in mediating adult sensitivity to heart failure (Mir133a-2) after in utero exposure to DEP.

Currently, the modulation of epigenetic biomarkers in utero is unclear, but it has been suggested that the presence of ROS can directly or indirectly affect DNA methylation, which results in persistent transcriptional changes [73]. In in vitro studies, 8-OHdG inhibits the methylation of the adjacent cytosines [74], a reaction that is catalyzed by DNA methyltransferases (DNMTs). Furthermore, 8-OHdG can influence the interaction of DNA with various transcription factors [75]. Although the mechanisms by which environmental stressors in utero alter the developing embryo and fetus are not clear. We suggest that the scenario caused by the in utero UFP exposure contributed to the fetal programming of RAS-related elements and altered blood pressure in the male offspring at adulthood. Recent studies on in utero exposure to particulate matter have shown oxidative stress, placental insufficiency [11], and cardiovascular susceptibility [10, 76], consistent with our findings. Furthermore, several experimental models of programming induced by gestational protein restriction [77, 78], maternal stress or placental insufficiency [79] have demonstrated that the induced hypertension is related to a marked increase in glucocorticoid expression and/or marked decrease in the expression of HSD11B2 [80].

## Conclusions

Our data suggested that the in utero exposure to UFP influenced adult susceptibility to increased blood pressure, which was influenced by alterations in the placenta and hypomethylation of the promoter region of RAS-related elements. The present study provided evidence of the consequence of air pollution exposure during development and gestation, where in utero exposure to UFP influenced the susceptibility to CVD in adulthood. These effects should be addressed in polluted cities as a public health issue.

## Additional files

Additional file 1: **Table S2.** Sex ratio and dam weight gain. (DOCX 41.2 kb)

Additional file 2: **Table S1.** DNA methylation analysis primer sequences. (DOCX 78 kb)
