# Supplementary material for: In utero exposure to ultrafine particles promotes placental stress-induced programming of renin-angiotensin system-related elements in the offspring results in altered blood pressure in adult mice
Source: Part Fibre Toxicol. 2019 Jan 28;16:7. doi: 10.1186/s12989-019-0289-1 (PMC6350404; doi:10.1186/s12989-019-0289-1)
Supplement: Supplementary file 1 — Table S2. Sex ratio and dam weight gain. (DOCX 41.2 kb) [file 12989_2019_289_MOESM1_ESM.docx]

**Table S2.** Sex ratio and dam weight gain.

|  | **CTRL (n=4)** | **H_2_O (n=4)** | **UFP (n=4)** |
| --- | --- | --- | --- |
| Sex ratio (mean ± SEM) | 53.20 ± 5.22 | 50.76 ± 6.20 | 51.88 ± 10.72 |
| Dam weight gain GD 17.5 (g) | 12.53 ± 1.16 | 13.33 ± 1.03 | 8.34 ± 0.77 * |

Sex ratio: male / (male + female) x 100.

* Statistically significant difference compared to the control and H_2_O group * p< 0.05. One-way ANOVA.
