# Supplementary material for: In utero exposure to ultrafine particles promotes placental stress-induced programming of renin-angiotensin system-related elements in the offspring results in altered blood pressure in adult mice
Source: Part Fibre Toxicol. 2019 Jan 28;16:7. doi: 10.1186/s12989-019-0289-1 (PMC6350404; doi:10.1186/s12989-019-0289-1)
Supplement: Supplementary file 2 — Table S1. DNA methylation analysis primer sequences. (DOCX 78 kb) [file 12989_2019_289_MOESM2_ESM.docx]

**Table S1.** DNA methylation analysis primer sequences.

| **Sequence name** | **Primer sequence (5’🡪3’)** | **Genomic location** | **Size product** |
| --- | --- | --- | --- |
| ***HSD11B2*** | F: GTAAGGTATAGGGTTGGGTAGATTG  R: [Btn]AAAACTTTCCTCCACTTCTATCTTAAC  S_1_: GTAAGGTATAGGGTTGGGTAGATTG  S_2_: GTAATTTGGGGATTTTGTTT | chr8:105,518,489-105,518,669 | 180 bp |
| ***Ace*** | F: TTATAGGGTTTTTTTTGTTTAGGG  R: [Btn]ACTACAACAACAACACCAACAAC  S_1_: TTATAGGGTTTTTTTTGTTTAGGG  S_2_: GGTGGTTGGGTTTTATAATT | chr11: 105,967,787-105,968,066 | 280 bp |
| ***Agtr1a*** | F: GAGTTTGGATTTGGAAGGGTATAT  R: [Btn]ATTCCAACCCTAAAACTTTACACTC  S: GAGTTTGGATTTGGAAGGGTATAT | chr:13: 30,336,237-30,336,383 | 147 bp |
| ***Agtr1b*** | F: TAGAATTTGTTAAGGGAGGGGTTA  R: [Btn]CTACCTAAAATCCAAACTACCTACAACTAT  S: TAGAATTTGTTAAGGGAGGGGTTA | chr3:20367157-20367310 | 161 bp |

F: forward, R: reverse and S: sequencing. Mouse genomic database (GRCm38/mm10), 2011.
